# Effect of orthodontic debonding and residual adhesive removal on 3D enamel microroughness

**DOI:** 10.7717/peerj.2558

**Published:** 2016-10-11

**Authors:** Joanna Janiszewska-Olszowska, Robert Tomkowski, Katarzyna Tandecka, Piotr Stepien, Tomasz Szatkiewicz, Katarzyna Sporniak-Tutak, Katarzyna Grocholewicz

**Affiliations:** 1 Department of General Dentistry, Pomeranian Medical University in Szczecin, Szczecin, Poland; 2 Faculty of Mechanical Engineering, Koszalin University of Technology, Koszalin, Poland; 3 Department of Technology and Education, Koszalin University of Technology, Koszalin, Poland; 4 Department of Maxillofacial Surgery, Pomeranian Medical University, Szczecin, Poland

**Keywords:** Orthodontic debonding, Orthodontic clean-up, Tungsten-carbide bur, One-step polisher and finisher, Adhesive residue remover

## Abstract

**Background:**

Termination of fixed orthodontic treatment is associated with bracket debonding and residual adhesive removal. These procedures increase enamel roughness to a degree that should depend on the tool used. Enamel roughening may be associated with bacterial retention and staining. However, a very limited data exists on the alteration of 3D enamel roughness resulting from the use of different tools for orthodontic clean-up.

**Aims:**

1. To perform a precise assessment of 3D enamel surface roughness resulting from residual adhesive removal following orthodontic debonding molar tubes.

2. To compare enamel surfaces resulting from the use of tungsten carbide bur, a one-step polisher and finisher and Adhesive Residue Remover.

**Material and Methods:**

Buccal surfaces of forty-five extracted human third molars were analysed using a confocal laser microscope at the magnification of 1080× and 3D roughness parameters were calculated. After 20 s etching, molar tubes were bonded, the teeth were stored in 0.9% saline solution for 24 hours and debonded. Residual adhesive was removed using in fifteen specimen each: a twelve-fluted tungsten carbide bur, a one-step finisher and polisher and Adhesive Residue Remover. Then, surface roughness analysis was repeated. Data normality was assessed using Shapiro–Wilk test. Analysis of variance (ANOVA) was used to compare between variables of normal distribution and for the latter—Kruskal-Wallis test.

**Results:**

Sa (arithmetical mean height) was significantly different between the groups (*p* = 0, 01326); the smoothest and most repeatable surfaces were achieved using Adhesive Residue Remover. Similarly, Sq (root mean square height of the scale-limited surface) had the lowest and most homogenous values for Adhesive Residue Remover (*p* = 0, 01108). Sz (maximum height of the scale-limited surface) was statistically different between the groups (*p* = 0, 0327), however no statistically significant differences were found concerning Ssk (skewness of the scale-limited surface).

**Discussion:**

Confocal laser microscopy allowed 3D surface analysis of enamel surface, avoiding the limitations of contact profilometry. Tungsten carbide burs are the most popular adhesive removing tools, however, the results of the present study indicate, that a one step polisher and finisher as well as Adhesive Residue Remover are less detrimental to the enamel. This is in agreement with a recent study based on direct 3D scanning enamel surface. It proved, that a one-step finisher and polisher as well as Adhesive Residue Remover are characterized by a similar effectiveness in removing residual remnants as tungsten carbide bur, but they remove significantly less enamel.

**Conclusion:**

Orthodontic debonding and removal of adhesive remnants increases enamel roughness. The smoothest surfaces were achieved using Adhesive Residue Remover, and the roughest using tungsten carbide bur.

## Introduction

Natural enamel microroughness is due to its microstructure. Enamel etching and resin infiltration into the superficial enamel layer during the bonding of orthodontic brackets makes it impossible to restore the original enamel condition after terminating fixed appliance therapy (Fjeld & Ogard, 2006). Bracket debonding and adhesive removal are associated with iatrogenic effects including: enamel cracking (Rix, Foley & Mamandras, 2001; Heravi, Rashed & Raziee, 2008; Dumbryte et al., 2013), enamel fracture (Zanarini et al., 2013; Janiszewska-Olszowska et al., 2014a; Janiszewska-Olszowska et al., 2014b), removing external enamel layer rich in fluoride (Al Shamsi et al., 2007; Banerjee et al., 2008; Ireland, Hosein & Sherriff, 2005; Hosein, Sherriff & Ireland, 2004; Pus & Way, 1980; Brown & Way, 1978; Fitzpatrick & Way, 1977; Janiszewska-Olszowska et al., 2015), leaving adhesive remnants (Janiszewska-Olszowska et al., 2014; Janiszewska-Olszowska et al., 2014b; Vieira et al., 1993; Ryf et al., 2012; Janiszewska-Olszowska et al., 2015) and surface roughening (Ahrari et al., 2013; Karan, Kiircelli & Tasdelen, 2010; Eliades et al., 2004; Roush et al., 1977). Adhesive remnants and surface roughening may be associated with plaque accumulation and discoloration (Joo et al., 2011). Moreover, the surface roughness of enamel and dental materials influences bacterial retention (Bollen, Lambrechts & Quirynen, 1997).

Few studies can be found describing enamel roughness following orthodontic debonding and clean-up. They were using contact profilometry (Ahrari et al., 2013; Eliades et al., 2004; Roush et al., 1997; Mahdavie et al., 2014; Faria-Junior et al., 2015), atomic force microscopy (Karan, Kiircelli & Tasdelen, 2010), rugosimetry (Cardoso et al., 2014) and 3D non-contact light profilometry (Ferreira et al., 2014). Two papers only have been found providing three-dimensional roughness parameters following orthodontic debonding from human enamel: one by Karan, Kiircelli & Tasdelen (2010) following adhesive removal with a tungsten carbide bur and a composite bur and the other by Ferreira et al. (2014) assessing the effect of different methods of enamel polishing following the use of tungsten carbide bur for adhesive removal.

It is obvious that enamel roughening during adhesive removal is dependent on the tool used. However, a very limited data exists on 3D roughness enamel alteration following the use of different tools, other than tungsten carbide bur. A recent study (Janiszewska-Olszowska et al., 2015) revealed that a one-step polisher and finisher as well as Adhesive Residue Remover have a lesser detrimental effect referring to enamel loss during adhesive removal than tungsten carbide bur. The aim of the present study was to perform a precise quantitative three-dimensional assessment of enamel surface roughness resulting from orthodontic debonding and adhesive removal and compare surfaces resulting from three different tools: tungsten carbide bur, a one-step finisher and polisher, and Adhesive Residue Remover.

## Material and Methods

This study has been approved by the bioethical committee of our university (decision reference No: KB-0012/35/16). An informed verbal consent was obtained from all participants.

In order to verify the sample size, an on-line power and sample size calculator was used (http://www.statisticalsolutions.net/calculators.php). The threshold value of clinical significance for both Sa and Sq values has been set at 0.5 (since Streptococcus mutans ranges in diameter from 0.5 to 0.75 μm). At the level of significance alfa = 0.05 and at the power of the test of 0.80, the sample size yielded 4.

Forty-five experimental teeth were selected from human third molars extracted for orthodontic reasons from patients aged 16–24 years (and stored hydrated at 4°C no longer than 2 weeks), basing on the criteria of intact buccal surfaces, lacks of carious lesions, restorations or visible cracks. They were stored in distilled water for 24 hours before bonding. Then, they were cleaned using a low speed bristle brush and non-fluoride pumice slurry, rinsed for 10 s and dried using oil-free compressed air.

For the purpose of confocal microscopy the roots were removed using a double-sided diamond disc. Then, the crowns were embedded in orthodontic plaster (Ortho Stone Extra White; Prevest Denpro GMBH, Heidelberg, Germany) with buccal surfaces exposed and numbered in sequence. After 20 s etching using 35% phosphoric acid (Ultra Etch; Ultradent) molar tubes were bonded directly with chemical-cure adhesive (3M Unitek; Unite) at the centre of the buccal surface, parallel to the crown long axis with slight pressure onto the enamel. Then, excess adhesive on the margins of the tubes was removed using a microbrush. Following 10 min setting, the specimen were stored in 0.9% saline solution for 24 hours, then rinsed with distilled water to prevent saline crystallization and debonded using ligature cutting pliers, positioned occlusally and gingivally to simulate the clinical conditions.

Then, the sample was divided into three groups (*n* = 15) according to the adhesive removing tool used. Three different tools were used for fifteen specimens each: a twelve-fluted tungsten carbide bur (123-603-00, Dentaurum, Pforzheim, Germany), a one-step finisher and polisher (inverted cone One gloss; Shofu Dental, Kyoto, Japan), and Adhesive Residue Remover (989-342-60; Dentaurum, Pforzheim, Germany). Clean-up procedure was performed under typical clinical conditions, by the same operator and continued until no macroscopically visible adhesive remnants could be found. Unfortunately, one of the specimens was accidentally damaged while using tungsten carbide bur and thus it had to be excluded from further assessment. The macroscopic amounts of the remaining adhesive were different for individual teeth, thus the authors decided not to assess the time needed to remove adhesive remnants.

**Figure 1:**
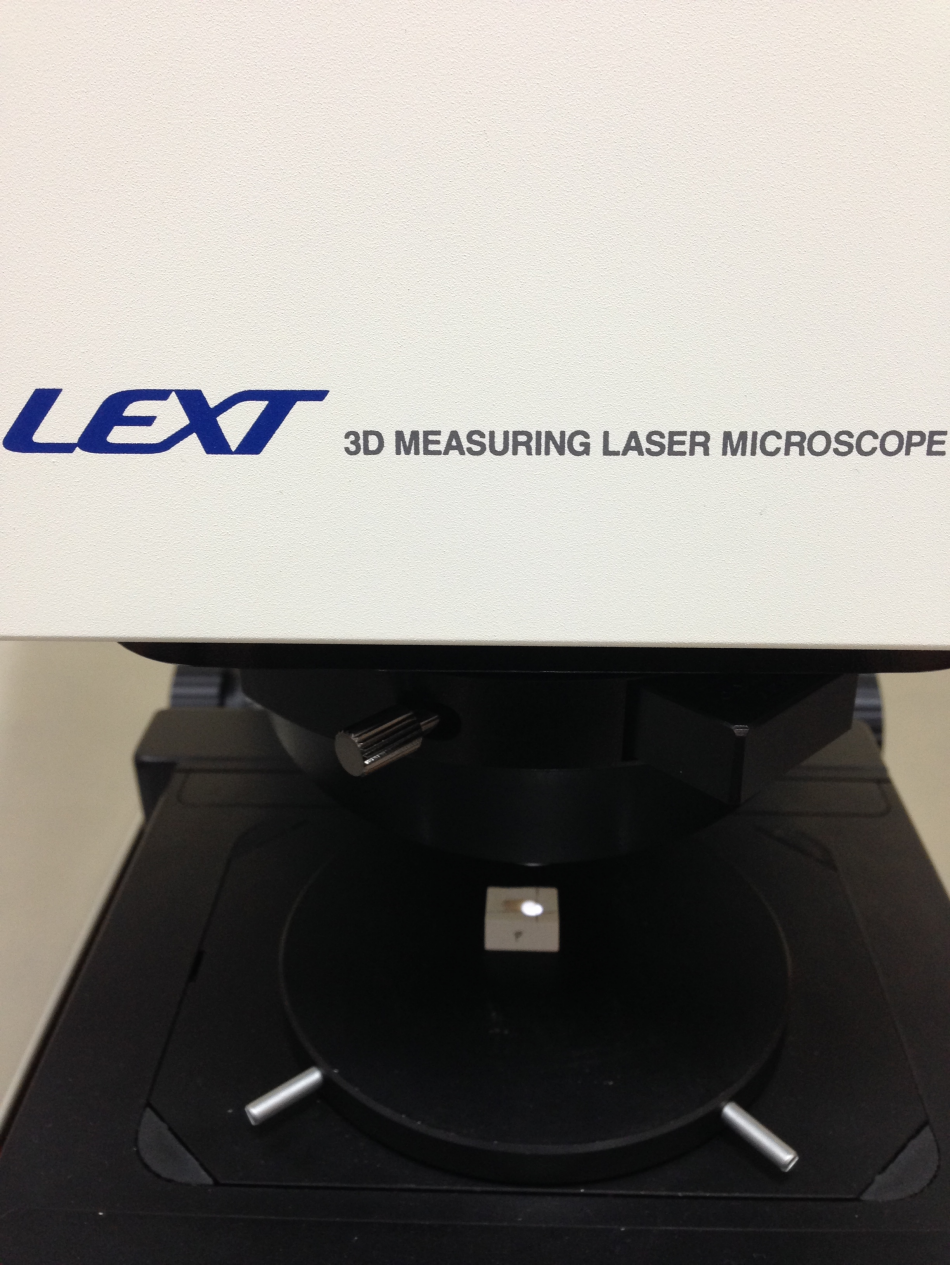
Enamel surface of a molar tooth (embedded in plaster) analyzed under confocal laser microscope.

Enamel roughness has been measured on labial enamel surfaces using laser confocal microscope (Lext OLS4000; Olympus, Tokyo, Japan) at the magnification of 1080× in the confocal mode using an area of observation of 256 μm × 256 μm (Fig. 1). Using the motorized table of the microscope the sample was aligned according to *x*, y and *z* coordinates from the marked starting point. Roughness measurement (confocal) mode was used to analyse the height information. The measuring units in *z*-axis were 10 nm. Laser microscope mode was used for the sample observation and acquiring images.

Measurements were performed before etching within the predicted bonding area in the central part of the buccal surface and at the same site after adhesive remnant removal. The author performing the measurement of enamel roughness and data processing was blinded to the tools and procedures used. Data processing has been performed using the specialized 3D analysis computer software—TalyMap Platinum 5.3 (Taylor-Hobson Ltd.). Data processing comprised surface levelling, non-measured points filling using a smooth shape calculated from the neighbours, shape (form) removal.

The following parameters according to ISO 25178-2 were used to describe enamel roughness:

Sa—arithmetical mean height,

Sq—root mean square height of the scale-limited surface,

Sz—maximum height of the scale-limited surface,

Ssk—skewness of the scale-limited surface.

Shapiro–Wilk test at the level *α* = 0.05 was used in order to check for data normality.

Analysis of variance (ANOVA) was used to compare between variables of normal distribution, whereas Kruskal-Wallis test was used for the latter.

## Results

Clinically, each of the tools used was able to remove all the visible adhesive remnants.

Shapiro–Wilk test revealed a normal distribution of the variables Sa and Sq for all the three tools as well as Sz for tugsten carbide bur. The results concerning initial enamel roughness have been presented in Table 1.

**Table 1:** Distribution of the roughness parameters: initial and following adhesive residue removal.

Tool used	Roughness parameter	Mean	SD	Median	Min	Max	Q1	Q3
None (initial values)	Sa (μm)	1,8902	0,35755974	0,8794	0,4743	1,9091	0,6659415	1,082215
	Sq (μm)	1,2375	0,475304583	1,0966	0,5933	2,4808	0,84803875	1,576215
	Sz (μm)	13,4611	6,86391067	11,0975	6,3622	39,9209	8,494445	14,876175
	Ssk	0,5389	1,046759739	0,2500	−0,5074	4,3566	0,003452025	0,529605
Tungsten carbide bur	Sa (μm)	1,0911	0,3257904	1,1852	0,3753	1,4561	0,9229155	1,3661975
	Sq (μm)	1,3867	0,379531851	1,4850	0,6367	1,9021	1,1640425	1,720065
	Sz (μm)	13,8126	1,857553128	13,7077	10,0807	16,7259	13,092875	15,05015
	Ssk	0,2981	1,232603685	0,0261	−0,5305	4,6519	−0,21576475	0,219953
Shofu One Gloss	Sa (μm)	0,8601	0,339741958	0,8542	0,3261	1,8118	0,693195	1,0039075
	Sq (μm)	1,1169	0,420468294	1,1180	0,5009	2,2693	0,8946345	1,363025
	Sz (μm)	14,1787	6,463893317	11,4921	6,9393	28,2629	10,44245	16,5144
	Ssk	0,4960	1,103817	0,0723	−0,6063	3,4260	−0,1944925	0,6799695
Adhesive residue remover	Sa (μm)	0,7521	0,174473783	0,7728	0,4443	1,0250	0,625502	0,873958
	Sq (μm)	0,9666	0,207533927	0,9448	0,5587	1,2705	0,8168535	1,15209
	Sz (μm)	13,4257	6,109329457	12,0051	6,7435	32,7994	9,904505	15,46585
	Ssk	0,3418	0,819283113	0,0452	−0,7620	2,6029	−0,0996192	0,96289

**Figure 2:**
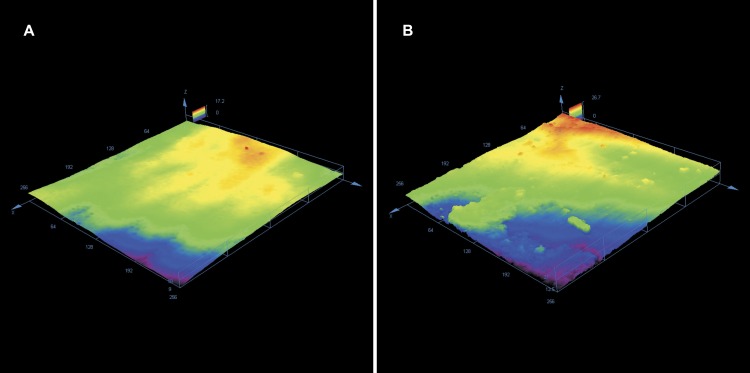
(A) Typical enamel surface before bonding, visible enamel prisms. (B) Following adhesive removal with tungsten carbide bur, visible surface roughening is present.

**Figure 3:**
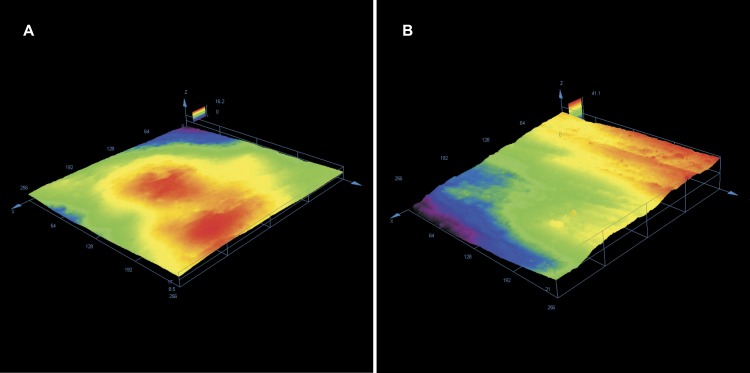
(A) Enamel surface before bonding with visible typical enamel surface topography. (B) Following adhesive removal using one-step finisher and polisher with visible scratching.

**Figure 4:**
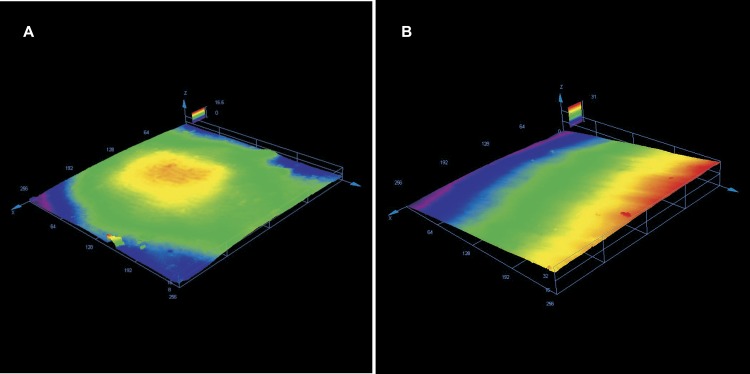
(A) Enamel surface before bonding with prisms visible. (B) Same surface following adhesive removal using Adhesive Residue Remover with visible changes in surface topography.

Enamel roughness parameters following adhesive rest removal have been presented in Table 1. Sa was significantly different between the groups (*p* = 0, 01326 at the level *α* = 0.05); the smoothest and most repeatable surfaces (lowest variance) were achieved using Adhesive Residue Remover, whereas the roughest surfaces were obtained by using tungsten carbide bur. Similar results were found concerning Sq (*p* = 0, 01108), which had the lowest and most homogenous values for Adhesive Residue Remover. Sz was statistically different between the groups (*p* = 0, 0327), however no statistically significant differences were found concerning Ssk. Typical enamel surfaces from each group of teeth before bonding and after orthodontic clean-up have been presented in Figs. 2–4.

## Discussion

Enamel surface roughness can be directly assessed on extracted human teeth only. Analysing epoxy replicas (Rouleau, Marshall Jr & Cooley, 1982; Krell, Courey & Bishara, 1993; Schuler & Van Waes, 2003; Sessa et al., 2012; Pont et al., 2010; Baumann, Brauchli & Van Vaes, 2011; Alessandri Bonetti et al., 2011) or silicone impressions (Fitzpatrick & Way, 1977) was used for visual assessment of enamel surface under SEM. They constitute indirect methods and do not allow a precise surface roughness measurement. However, it should be remembered that the experimental conditions may differ from an *in vivo* situation, especially referring to a better visual control of enamel surface during manipulation *in vitro* (Faria-Junior et al., 2015). No correlation has been found between the amount of adhesive remnants and scoring of enamel surface after debonding and clean-up (Pont et al., 2010). Thus, no analysis of adhesive remnants before clean-up was performed in the present study.

An often cited critical threshold surface roughness for bacterial adhesion of 0.2 μm has been established by Quirynen et al. (1990); Quirynen, Papaioannou & Steenberghe (1996) who found that bacterial accumulation increases above this value and does not reduce with its decrease. However, it should be remembered, that these studies were not performed on enamel surface, but on resin strips and implants, which are artificial materials. The enamel surface is far more complex—the presence of waviness, pits, fissures and other irregularities—allows for easier bacterial colonization protected from shear forces (Bollen, Lambrechts & Quirynen, 1997). Thus these straight rules may not apply.

The method of laser scanning confocal microscopy is free from the limitations of a contact (stylus) profilometry. Contact surface roughness measuring devices cannot measure micro asperities less than the stylus tip diameter, moreover they can be damaged by surface wear. The confocal laser microscope observation allowed to avoid sample sputtering, thus enamel surface could be analyzed before etching and after adhesive remnant removal. The analysis of a surface in 3D is more reliable than of a single profile. Sa is a surface parameter, and for technical surfaces the relationship between Ra and Sa is 1.25; however, this rule does not have to apply to biological specimen. It should be kept in mind that measured roughness parameters of natural surfaces are influenced by the measurement device and magnification. The higher the level of magnification, the lower Ra or Sa values measured for the same surface. Thus, the results from various studies cannot be easily compared and no study reporting human enamel 3D roughness parameters measured at a similar magnification has been found for comparisons.

High values of the roughness parameters that appeared different between the groups of teeth (arithmetical mean height of the surface, root mean square height of the surface and maximum height of the surface) describe a rough surface with high “peaks” and deep “voids”, reflecting the severity of mechanical damage. No difference concerning skewness reflects random shapes of the scratches.

Numerous studies describe the use of tungsten carbide burs for adhesive removal (Rouleau, Marshall Jr & Cooley, 1982; Zarrinnia, Eid & Kehoe, 1995; Campbell, 1995; Retief & Denys, 1979; Gwinnett & Gorelick, 1977). Equal results concerning enamel roughness resulting from the use of 12-, 16- and 20-fluted carbide burs were reported (Webb et al., 2016). Thus different types of burs were not analyzed in the present study. A recent study (Janiszewska-Olszowska et al., 2015) comparing tungsten carbide bur, one-step finisher and polisher and Adhesive Residue Remover proved their similar effectiveness in adhesive removal. The volume of adhesive remnants measured on direct 3D scans after enamel clean-up did not differ signficantly between the tools used. However, the amount of enamel removed was highest when using tungsten carbide bur and lowest—when using Adhesive Residue Remover.

The one step polisher and finisher used—One Gloss is a rubber wheel, which employs aluminium dioxide and silicone dioxide as an abrasive and the delivery medium for abrasive is polyvinylsiloxane (Yap et al., 2004). Polyvinylosiloxane is elastic and may be resistant to wear by fillers of adhesive resins. According to the manufacturers, one-step polishing and finishing systems have been introduced in order to reduce costs and chair-time. They use altered pressure instead of varied size of abrasive particles.

The first study found describing the use of an elastic tool for orthodontic adhesive removal is by Gwinnett & Gorelick (1977), who have described orthodontic adhesive removal by a green rubber wheel. They found this elastic rotary tool more effective than green stone, white stone, sandpaper discs, tungsten carbide bur, steel bur or acrylic steel bur. Green rubber wheel has been assessed as less destructive than the most popular tungsten carbide burs. It provided a macroscopic polish and under microscope there were fine scratches visible, which could be removed with pumice prophylaxis paste. However, it should be kept in mind, that these results were based on visual enamel surface assessment under SEM, not on instrumental measurements. No later studies reporting the use of green rubber wheel could be found.

The Adhesive Residue Remover, a stiff rotary tool, made of epoxy resin and glass, proved to leave the smoothest and most predictable surfaces. This is consistent with the results by Janiszewska-Olszowska et al. (2015) basing on 3D scanning of enamel surface, where Adhesive Residue Remover removed the least amount of enamel compared to tungsten carbide bur and one-step finisher and polisher.

In the present study molar tubes were removed from enamel similarly as in the clinical condidtions, thus a cumulative effect of debonding and adhesive removal has been analysed. In the study by Ahrari et al. (2013), the brackets were isolated from adhesive by a layer of vaseline, thus no enamel defects could result from debonding (bond failure between adhesive and enamel).

The present study reports enamel roughness following adhesive removal, but before polishing. From the study by Eliades et al. (2004), it can be expected that the parameters of roughness: Ra, Rt and Rz are not influenced by polishing, only Rq representing height distribution to baseline reduced after polishing. This is due to the fact, that grooves produced by adhesive removing tools remain after polishing—but height is reduced by removing material from peak surface. These findings support some statements, based on qualitative enamel surface rating under SEM, that enamel scratching caused by adhesive removing tools is not removed by polishing (Vieira et al., 1993; Gwinnett & Gorelick, 1977). Similarly, Roush et al. (1977) and Ahrari et al. (2013) stated, that final polishing failed to restore enamel roughness to pretreatment values. Keeping this in mind, it should be considered important to minimize enamel roughening caused by orthodontic adhesive removal.

Unfortunately, no tools exist allowing analyze enamel surface roughness *in vivo*. Thus a visually assessed reduction in enamel irregularities at 6 and 12 months follow-up found in SEM observation of enamel surface replicas (Gracco et al., 2015) cannot be confirmed in instrumental measurements.

## Conclusion

(1) Removal of orthodontic adhesive remnants increases enamel roughness to a various degree, depending on the tool used.
(2) The smoothest surfaces were achieved using Adhesive Residue Remover and the roughest –using tungsten carbide bur.

##  Supplemental Information

10.7717/peerj.2558/supp-1 Data S1 Roughness parameters before bonding Initial Sa, Sq, Sz and Ssk for every tooth analyzed.

10.7717/peerj.2558/supp-2 Data S2 Roughness parameters following adhesive removal Sa, Sq, Sz and Ssk following the use of three different tools for adhesive residue removal.

## Additional Information and Declarations

### Data Availability

The following information was supplied regarding data availability: The raw data has been supplied as Supplemental Files.
