# Supplementary material for: Effect of orthodontic debonding and residual adhesive removal on 3D enamel microroughness
Source: PeerJ. 2016 Oct 11;4:e2558. doi: 10.7717/peerj.2558 (PMC5068349; doi:10.7717/peerj.2558)
Supplement: Data S2 — Sa, Sq, Sz and Ssk following the use of three different tools for adhesive residue removal. [file peerj-04-2558-s002.docx]

Table enamel roughness following adhesive removal

| Tool used | Parameter of roughness | Sa | Sq | Sz | Ssk |
| --- | --- | --- | --- | --- | --- |
|  | Tooth number |  |  |  |  |
| Tungsten carbide bur | **1** | 1,4248 | 1,7539 | 13,4472 | 0,2338 |
|  | **2** | 0,6757 | 0,8718 | 13,3619 | 0,3038 |
|  | **4** | 0,9032 | 1,1376 | 16,2835 | 0,1502 |
|  | **15** | 1,2887 | 1,5727 | 13,0032 | -0,3434 |
|  | **21** | 0,9821 | 1,2489 | 11,7143 | -0,2477 |
|  | **22** | 1,4330 | 1,7903 | 14,3054 | 0,1785 |
|  | **24** | 1,3217 | 1,6186 | 13,7884 | 0,1770 |
|  | **25** | 0,3753 | 0,6367 | 13,6270 | 4,6519 |
|  | **30** | 1,3810 | 1,7835 | 16,7259 | -0,0980 |
|  | **31** | 1,2713 | 1,5403 | 10,0807 | -0,1964 |
|  | **33** | 1,4561 | 1,9021 | 16,1826 | 0,2537 |
|  | **36** | 0,9879 | 1,2435 | 14,2196 | -0,1371 |
|  | **37** | 1,0992 | 1,4296 | 15,2984 | -0,5305 |
|  | **49** | 0,6752 | 0,8839 | 11,3378 | -0,2222 |
|  | **15 damaged** |  |  |  |  |
| Shofu One Gloss | **5** | 0,4596 | 0,5718 | 6,9393 | -0,3341 |
|  | **6** | 0,3261 | 0,5009 | 10,8707 | 3,4260 |
|  | **12** | 0,8542 | 1,1180 | 11,4921 | 0,1161 |
|  | **16** | 0,8618 | 1,1549 | 12,2404 | -0,3888 |
|  | **17** | 1,1168 | 1,3879 | 16,8563 | 0,0485 |
|  | **18** | 0,8676 | 1,3382 | 28,2629 | 2,4479 |
|  | **20** | 0,9283 | 1,2478 | 21,3233 | 1,4603 |
|  | **26** | 0,7067 | 0,9138 | 10,8831 | -0,6063 |
|  | **27** | 1,8118 | 2,2693 | 16,1725 | 0,3838 |
|  | **34** | 1,1672 | 1,4029 | 9,4040 | 0,0723 |
|  | **40** | 0,5823 | 0,7002 | 6,9547 | -0,0549 |
|  | **42** | 1,0795 | 1,4060 | 27,4567 | 0,3302 |
|  | **44** | 0,7299 | 0,9290 | 10,7914 | -0,4880 |
|  | **46** | 0,6797 | 0,8755 | 10,0935 | 0,0511 |
|  | **50** | 0,7304 | 0,9368 | 12,9402 | 0,9761 |
| Adhesive residue Romover | **3** | 0,7535 | 0,9308 | 32,7994 | -0,0687 |
|  | **7** | 0,7728 | 0,9855 | 9,8936 | -0,7620 |
|  | **8** | 0,6381 | 0,8238 | 12,4689 | -0,3598 |
|  | **9** | 0,4713 | 0,6920 | 11,2268 | 2,6029 |
|  | **10** | 1,0242 | 1,2375 | 9,2524 | 0,0897 |
|  | **13** | 1,0250 | 1,2705 | 15,9745 | 0,0296 |
|  | **14** | 0,9333 | 1,2249 | 16,3326 | 0,9431 |
|  | **19** | 0,8115 | 1,0617 | 14,9572 | 1,1145 |
|  | **35** | 0,6625 | 0,8896 | 10,4917 | -0,4330 |
|  | **38** | 0,4443 | 0,5587 | 6,9355 | -0,1103 |
|  | **39** | 0,7760 | 0,9448 | 9,9155 | -0,0889 |
|  | **41** | 0,8169 | 1,1452 | 14,3887 | 0,9827 |
|  | **45** | 0,9310 | 1,1590 | 18,0009 | 0,1447 |
|  | **47** | 0,6088 | 0,8099 | 12,0051 | 0,9971 |
|  | **48** | 0,6129 | 0,7644 | 6,7435 | 0,0452 |
